# Low-power flexible organic memristor based on PEDOT:PSS/pentacene heterojunction for artificial synapse

**DOI:** 10.3389/fnins.2022.1016026

**Published:** 2022-09-08

**Authors:** Xiliang Luo, Jianyu Ming, Jincheng Gao, Jingwen Zhuang, Jingwei Fu, Zihan Ren, Haifeng Ling, Linghai Xie

**Affiliations:** State Key Laboratory of Organic Electronics and Information Displays and Institute of Advanced Materials (IAM), Nanjing University of Posts and Telecommunications, Nanjing, China

**Keywords:** memristor, low power, flexible, PEDOT:PSS, artificial synapse

## Abstract

Organic synaptic memristors are of considerable interest owing to their attractive characteristics and potential applications to flexible neuromorphic electronics. In this work, an organic type-II heterojunction consisting of poly(3,4-ethylenedioxythiophene): polystyrene sulfonate (PEDOT:PSS) and pentacene was adopted for low-voltage and flexible memristors. The conjugated polymer PEDOT:PSS serves as the flexible resistive switching (RS) layer, while the thin pentacene layer plays the role of barrier adjustment. This heterojunction enabled the memristor device to be triggered with low-energy RS operations (*V* < ± 1.0 V and *I* < 9.0 μA), and simultaneously providing high mechanical bending stability (bending radius of ≈2.5 mm, bending times = 1,000). Various synaptic properties have been successfully mimicked. Moreover, the memristors presented good potentiation/depression stability with a low cycle-to-cycle variation (CCV) of less than 8%. The artificial neural network consisting of this flexible memristor exhibited a high accuracy of 89.0% for the learning with MNIST data sets, even after 1,000 tests of 2.5% stress-strain. This study paves the way for developing low-power and flexible synaptic devices utilizing organic heterojunctions.

## Introduction

Artificial synapses are attracting increased attention as the key in-memory computational cell for neuromorphic computing (Zhu et al., 2020; Gao et al., 2021; Wang et al., 2022). Resistive switching (RS)-based memristor is being actively investigated as an artificial synapse due to its low power consumption, tunable conductance, and high scalability (Xia and Yang, 2019; Kumar et al., 2022). Memristors are normally in the form of a metal–dielectric–metal two-terminal structure, in which the RS materials allow for tunable resistance states while applying an external voltage. A wide variety of organic, inorganic, and organic–inorganic hybrid materials have been found to display RS property. Among them, transition–metal oxide materials are being intensively researched because they allow for large scale system integration in CMOS process (Cao et al., 2017; Sun et al., 2018; Wang et al., 2018; Yao et al., 2020). On the other hand, with the widespread application of wearable electronics, large amount of unstructured data is putting high demands on the data preprocessing capability of portable devices in real time, leading to the requirement for low power flexible memristors (Goswami et al., 2020; Tao et al., 2020). Due to the limitation of Young’s modulus, however, the development of flexible memristors using transition–metal oxide materials remains a challenge.

Owing to the tailorable electronic and optical properties, and intrinsic flexibility, organic material has emerged as a promising RS medium for flexible memristor (Lim et al., 2015; Wehner et al., 2016; Kim et al., 2018; Must et al., 2019; Wang et al., 2020). Conductive polymer poly (3,4-ethylene dioxythiophene): poly (styrene sulfonate) (PEDOT: PSS) with tunable electrical conductivity and high flexibility, permeability, has been widely studied in neuromorphic devices. Serving as the temporary reservoir or transport path of abundant inflow ions, PEDOT: PSS could incorporate alkali metal ions or halide ions to build different diffusive or drift kinetics (Feng et al., 2019; John et al., 2021). Besides, PEDOT:PSS could also offer a physical barrier to migrate ions to prevent abrupt RS that benefits analog switching (Xiao and Huang, 2016; Yang J. M. et al., 2021). However, a voltage bias over 1 V is always required to operate these memristor device (Choi et al., 2017; John et al., 2018). Since both the conductivity and charge injection barrier play a significant role in determining the operation voltage, investigation of charge transport layers would enable further insights into the designing of low power memristors.

In this work, low-power flexible organic memristor using PEDOT:PSS/pentacene heterojunction as RS core layers was proposed. The electrical conductivity and mechanical flexibility of the PEDOT:PSS film were enhanced by the addition of additives. A type-II heterojunction was formed by introducing a thin pentacene layer together with PEDOT:PSS to decrease the charge injection barrier. A low operating voltage of 1.0 V was achieved to trigger analog RS properties. The device successfully emulated potentiation and depression, spike-rate-dependent plasticity (SRDP), and spike-duration-dependent plasticity (SDDP), etc., according to applied voltage pulses. Besides, the memristors presented low cycle-to-cycle variation (CCV) during the update of the conductance states, enabling the high recognition accuracy of 92.6% for handwriting digit recognition. The neural network training process of the flexible device after mechanical bending was further studied, and the recognition accuracy of 89.0% can still be achieved under a nearly folded bending radius (2.5 mm), revealing the potential for flexible electronic applications.

## Results and discussion

### Analog resistive switching properties

**FIGURE 1:**
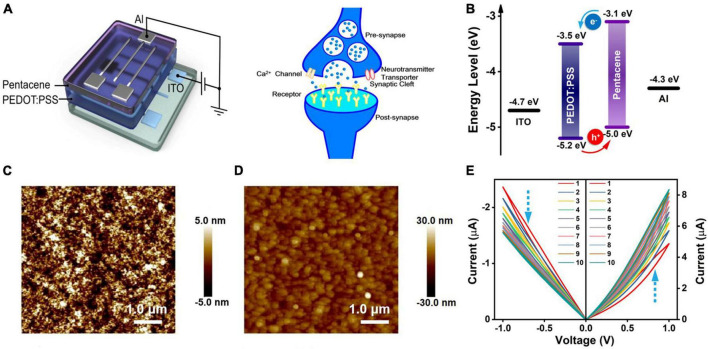
**(A)** Device schematic of ITO/PEDOT:PSS/pentacene/Al memristor and schematic illustration of a biological synapse. **(B)** Energy levels of the materials used in the memristor device. **(C)** AFM image of PEDOT:PSS thin film on ITO substrate. **(D)** AFM image of pentacene thin film on PEDOT:PSS film. **(E)** *I-V* characteristics of the memristor device under a linear voltage scan with the step size of 0.05 V (0 to 1.0 V and 0 to –1.0 V).

Figure 1A showed the schematic illustration of the ITO/PEDOT:PSS/pentacene/Al memristor (left) and a biological synapse (right). All electrical characteristics were carried out with the top Al electrode grounded. In biological synapse, information is transmitted by neurotransmitter release in the cleft between the pre-and post-synaptic neurons via calcium ion influx (Kim et al., 2019; Seo et al., 2020). The ITO bottom electrode (BE) and the Al top electrode (TE) acted as pre-and post-synaptic neurons. The hole transport layer of PEDOT:PSS was spin coated onto ITO from dispersions containing additives ethylene glycol (EG, conductivity enhancing agent), dodecyl benzene sulfonic acid (DBSA, surfactant) and (3-glycidyloxypropyl) trimethoxysilane (GOPS, crosslinking agent), resulting in a *ca.* 80-nm-thick RS film (Supplementary Figure 1). As illustrated in Figure 1B, the hole injection barrier (0.5 eV) between ITO and PEDOT:PSS is much smaller than the electron injection barrier (1.2 eV) between PEDOT:PSS and Al, indicating that hole carriers dominate the injection from ITO to the highest occupied molecular orbital (HOMO) level of PEDOT:PSS (Khan et al., 2019; Wang et al., 2021). A 9-nm-thin pentacene film was chosen as the *p*-type interlayer between PEDOT:PSS and Al. Pentacene showed a higher HOMO level (−5.0 eV) than that of PEDOT:PSS (−5.2 eV), forming a “staggered-gap” type-II heterojunction with PEDOT:PSS, thus further facilitating hole conduction (Lee et al., 2008; Kim et al., 2012; Dong et al., 2013). Besides, the atomic force microscopy (AFM) images shown in Figure 1C revealed that PEDOT:PSS film contained uniformly distributed domains, indicating the formation of a crystallized PEDOT:PSS film. The crystallization originated from the PEDOT chains’ aggregation due to the addition of polyhydroxy alcohols, which increased the film conductivity (Zhang et al., 2014; Ouyang et al., 2015). Moreover, since the interfaces play a significant role in defining the charge injection barriers, the surface morphology of pentacene was also investigated. As shown in Figure 1D, the pentacene layer presented numerous and small crystalline grains. Numerous nucleation sites for the growth of pentacene in the thermal evaporation process were attributed to the crystalline characteristics of PEDOT:PSS film, meanwhile the ultra-slow deposition rate (0.05–0.1 Å/s) to form 9-nm film also contributed to the formation of small and numerous grains (Pratontep et al., 2004; Loi et al., 2005). These nanostructures would provide a large interfacial area for hole conduction between PEDOT:PSS and pentacene (Ling et al., 2016).

Figure 1E showed the current-voltage (*I*-*V*) characteristics of the as fabricated device under ten times of successive positive (0 V → 1.0 V → 0 V) and negative (0 V → –1.0 V → 0 V) dual voltage sweeps. The memristor exhibited gradual increasing/decreasing change of the currents in positive/negative voltage sweeping, respectively, indicating an analog RS property and the synaptic weight potentiation and depression. The operation current was lower than 10 μA, demonstrating a low power consumption. In the positive dual voltage sweeps, the memristor device showed counterclockwise hysteresis behaviors, indicating that a high-resistance state (HRS) for the forward sweep changed to a low-resistance state (LRS) for the reverse sweep after 1.0 V. Therefore, the device current gradually increased with increasing number of voltage dual sweep. In the negative dual voltage sweeps, however, the initial LRS changed to a HRS after –1.0 V, resulting in a gradual decrease of device current caused by 10 consecutive negative dual voltage sweeps. Moreover, the *I–V* characteristics showed rectifying behaviors, with the current measured in positive voltage region higher than that in negative voltage region. This behavior was attributed to the relatively higher electron injection barrier. Although pentacene provided an ohmic contact to PEDOT:PSS while having a sufficiently high LUMO to function as an electron-blocking layer. However, the rectifying property could effectively mitigate the sneak path currents in a large crossbar array. When the sweeping voltage was further increased to ± 2.0 V (Supplementary Figure 2), the current value of LRS was much higher and the dynamic range of LRS/HRS was larger, suggesting more holes were injected. Controlling devices of ITO/PEDOT:PSS/Al and ITO/pentacene/Al were also fabricated to further probe the RS mechanism. The pure PEDOT:PSS device exhibited disordered and poor RS curves until a high voltage of 6.0 V was applied (Supplementary Figure 3). And the pure pentacene device showed no RS characteristic even when a high voltage of 10.0 V was applied. Furthermore, the PEDOT:PSS heterojunction-based device without additives was investigated (Supplementary Figure 4), exhibiting a higher operating voltage (± 4.0 V). The lower conductivity of PEDOT:PSS thin film should be responsible for this high operating voltage.

Therefore, the organic memristors having a bilayer structure composed of a thick conductive polymer layer and a thin barrier adjustment layer, where the modulation of interfacial barrier depending on device operation history has an impact on device resistance state and results in the memristive characteristics. Initially, the device is in the off-state with PEDOT^0^ acting as the charge-trapping site (Wang et al., 2010). When a positive voltage was applied on the ITO electrode, holes were injected into the PEDOT film and PEDOT^0^ was oxidized into PEDOT^+^ (PEDOT^0^ + h^+^ = PEDOT^+^). With increased positive voltages, the charged PEDOT^+^ leaded to the accumulation of space charges and decreased the interface barrier. In this case, the memristor resistance gradually changed from HRS to LRS. By the contrast, the PEDOT^+^ would be reduced to PEDOT^0^ (PEDOT^+^ + *e*^–^ = PEDOT^0^) when a negative voltage was applied and hence, changed the memristor from LRS to HRS.

Furthermore, the conduction mechanism of PEDOT:PSS/pentacene heterojunction-based memristor could be explained by Ohmic conduction and Space-charge-limited conduction (SCLC) (Supplementary Figure 5). The first voltage loop of *I–V* curves in dual-logarithmic scales was plotted to interpret the conduction mechanism. In the forward low voltage region (0 to 0.9 V), the relation of current and voltage satisfied the behavior of Ohmic conduction (*I*∝*V*) with a fit slope of about 1.08. The PEDOT:PSS polycrystalline film served as the charge-trapping site, while the weak electric field could not effectively inject the charges to oxidize the PEDOT^0^ to PEDOT^+^. When the voltage is larger than trap filled limit voltage (*V*_*TFL*_) of 0.9 V, the current increased rapidly with a slope of about 2.66, suggesting the electric field across the device is sufficient and all trap sites are filled with charge carriers, thus the device finished the transition from Ohmic conduction to SCLC. And the SCLC could be expressed as the following formula:


(1) $$I\propto V^{n}$$

Where *n* is the exponential parameter related to the charge trapping state, and *n* would decrease for gradually retentive trapping charges in the RS layer (Wang et al., 2017a). At the backward voltage in the positive voltage region, the dual-logarithmic curve exhibited a lower slope as the applied voltage decreased. However, a current drop at *V*_*TFL*_ does not appear, which indicated trapping charges still be maintained below the *V*_*TFL*_, especially the retentive deep ones, resulting in clear hysteresis in the positive bias region (Wang et al., 2017b). As the voltage decreases further (0.65 to 0 V), the thermionic emission charge dominated the device and back to Ohmic conduction. In negative voltage region, the Ohmic conduction and Space-charge-limited conduction are still available (Supplementary Figure 5B). But relatively higher electron injection barrier (0.4 eV) led to a higher *V*_*TFL*_ (1.4 V) in forward sweeping, corresponding to a low exponent (*n*≈1.83) of SCLC process, thus ultimately obtaining a smaller hysteresis loop area and asymmetry of the *I–V* curves in different polarities.

### Synaptic plasticity functions

**FIGURE 2:**
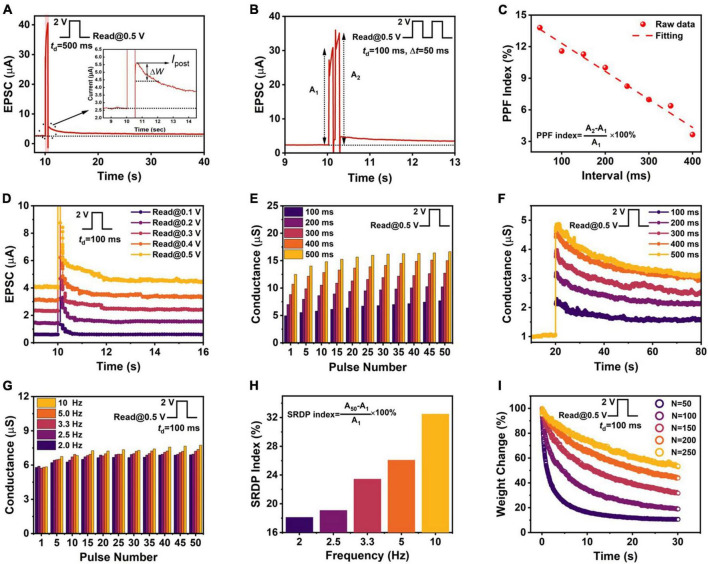
**(A)** EPSC characteristics triggered by positive voltage pulse (*V*_*p*_ = 2 V, *t*_d_ = 500 ms). **(B)** PPF behavior triggered by a pair of positive voltage pulses (*V*_P_ = 2 V, *t*_d_ = 100 ms, Δ*t* = 50 ms). **(C)** PPF index plotted as a function of pulse interval. **(D)** EPSC generated by a sequence of positive voltage pulse with different read voltages. **(E)** SDDP characteristics triggered by different pulse durations. **(F)** Conductance decay behavior after the positive voltage pulse with different durations. **(G)** SRDP characteristics triggered by different pulse frequencies. **(H)** SRDP index plotted as a function of pulse frequency. **(I)** Pulse-number-dependent Δ*W* (*I*_s_ read at 30 s).

In a synaptic memristor, the conductance can be regarded as a synaptic weight. The conductance change was represented by the post-synaptic current (PSC). As presented in Figure 2A, when a positive voltage pulse (*V*_*P*_ = 2.0 V, *t*_*d*_ = 500 ms) was applied, the device current increased from 2.6 μA (resting-state current, *I*_*r*_) to 40.7 μA, indicating the excitatory post-synaptic current (EPSC). After the short positive voltage pulse, the inset of Figure 2A showed that EPSC decayed from 5.6 μA to a stable value of 3.2 μA after a relaxation time of nearly 23.5 s. This decay was ascribed to the recombination of the holes and electrons, and thus the amount of space charge accumulation decreased. However, the device conductance changed to a quasi-permanent and higher level of resting state (3.2 μA), suggesting a modification of synaptic strength after stimulation. EPSC could also be triggered by a pulse fixed at 1.0 V (Supplementary Figure 6). But even with the maximum duration (*t*_*d*_ = 500 ms) stimulation, the device current could only change from 0.56 μA to the quasi-permanent resting state of 0.66 μA. At a small voltage (1.0 V), relatively fewer charges are injected into the PEDOT:PSS film, resulting in fewer charges maintaining the trapped state after relaxation time (23.5 s). Furthermore, single synaptic event energy consumption was obtained by decreasing the pulse width (10 ms) and amplitude (1 V), and a low energy consumption was estimated to be 2,740 fJ μm^–2^ (Supplementary Figure 7).

Paired-pulse facilitation (PPF) is one of the important forms of short-term plasticity (STP) behavior (Santschi and Stanton, 2003). Two correlated pulses simulation with different interval time (Δ*t*) were applied to the ITO BE and produced paired current peaks (*A*_1_ and *A*_2_). As displayed in Figure 2B, the peak current triggered by the second electrical signal pulse (*A*_2_) was larger than that triggered by the first electrical signal pulse (*A*_1_). In the PEDOT:PSS based artificial synapse, the behavior of PPF can be explained by the accumulation of charged PEDOT^+^ in the thick PEDOT:PSS layer when the interval time between two pulse stimulations is shorter than the recombination time of holes and electrons in the device, which enhanced the device conductance. The index of PPF can be expressed as the following equation:


(2) $$index_{PPF}=\frac{A_{2}-A_{1}}{A_{1}}\times 100\%$$

As the pulse interval increased (Figure 2C), the PPF index decreased to approach the value of 0, indicating the temporal correlations between the two neurons were gradually lost. When paired negative pulses were applied to ITO BE (Supplementary Figure 8), the inhibitory postsynaptic current (IPSC) and paired-pulse depression (PPD) behaviors could also be triggered in this memristor due to the reduction of PEDOT^+^. Taking into account the effect of read voltage for low operating voltage memristors, the synaptic weight change (Δ*W*) was used for comparison. The resting-state current (*I*_*r*_) before stimulation and the current read at a specific time after stimulation (*I*_*s*_, read at 16 s in this case) were selected for calculation, as shown in the following equation:


(3) $$\Delta W=\frac{I_{s}-I_{r}}{I_{r}}\times 100\%$$

As shown in Figure 2D, when the read voltage increased from 0.1 to 0.5 V, the level of *I*_*r*_ increased, and the Δ*W* increased from −2.9 to + 10.8%. We deduced that a small read voltage could slightly drive a few numbers of holes to move to the interface, biasing the device into a sub-stable state. When a larger stimulus voltage pulse came, there will be more holes to be injected as well as better modulation of the synaptic weight (Ling et al., 2019a). After the voltage pulse stimulation, the time required for the EPSC to decrease from maximum post-synaptic current value (*I*_*post*_, 100%) to 60% was defined as the relaxation time (*t*_*r*_). The *t*_*r*_ increased from 300 to 1,200 ms when the read voltage increased from 0.1 to 0.5 V, indicating the implementation of STP to long-term plasticity (LTP) transition. Hence 0.5 V was selected as the read voltage in the subsequent operation.

Figures 2E,F showed that when the pulse duration *t*_*d*_ increased, an obvious enhancement in device conductance was observed along with a prolonged *t*_*r*_, which was regarded as spike-duration-dependent plasticity (SDDP). As the number of pulses and pulse duration increased simultaneously, the device conductance showed a rapid increase for the first 20 pulses and then reached saturation. This can be ascribed to those previously accumulated space charges that could form a built-in electric field and block subsequent hole injection. Spike-rate-dependent plasticity (SRDP) is also one of the most important synaptic properties and underlies the brain’s establishment of spatiotemporal information processing (Abbas et al., 2020). Figure 2G illustrated the conductance change by applying 50 V pulses with a fixed amplitude of 2.0 V of different frequencies (2.0, 2.5, 3.3, 5.0, and 10.0 Hz). The peak current was ramped up in response to the increase of pulse rate. The SRDP index was plotted with respect to frequency, as shown in Figure 2H. The peak current triggered by the fiftieth (first) voltage pulse was deemed as *A*_50_ (*A*_1_). The SRDP index can be expressed as the following equation:


(4) $$index_{SRDP}=\frac{A_{50}-A_{1}}{A_{1}}\times 100\%$$

The SRDP index increased from 18.1 to 32.5% when the frequency of pulses applied was increased from 2.0 to 10.0 Hz, providing the possibility for application to dynamic filter (Sun et al., 2019). For exploring the relationship between the number of repetitive stimuli and Δ*W*, the pulses with fixed voltage pulse were applied (*V*_*P*_ = 2.0 V, *t*_*d*_ = 100 ms). Figure 2I showed that when the repetitive times increased from 50 to 250, the Δ*W* increased from 10.8 to 53.8%, indicating a repetitive stimulus is an effective way to build connections between synapses.

### Simulation of artificial neural network

**FIGURE 3:**
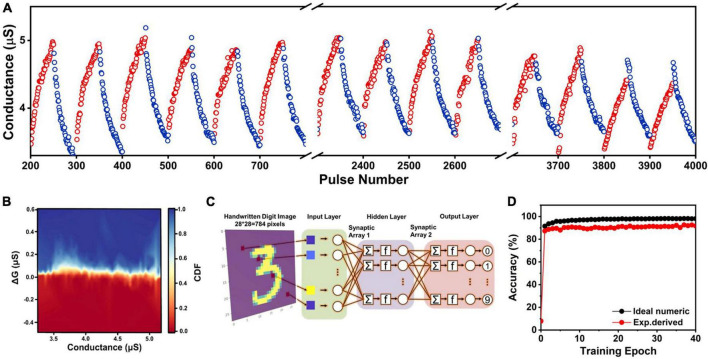
**(A)** Potentiation-depression characteristics of the memristor device. Pulsing scheme: 50 pulse training cycles, with each pulse training cycle comprising 50 identical positive pulses (*V*_P_ = 2.0 V, *t*_d_ = 100 ms, *V*_read_ = 0.5 V) and 50 identical negative pulses (*V*_D_ = –1.5 V, *t*_d_ = 100 ms, *V*_read_ = 0.5 V) with 100 ms pulse interval. **(B)** Δ*G* vs. *G* switching statistics of memristor device during potentiation. **(C)** Artificial neural network for learning MNIST data based on MLP model. **(D)** The recognition accuracy evolution with training epochs for handwritten digit image.

In a memristor array, the vector matrices are represented by the conductance matrices of the synaptic devices, where the weight value maps to the conductance of the device. The weight update behaviors were investigated using identical pulse scheme. A training cycle contained 50 positive voltage pulses (*V*_*P*_ = 2.0 V, *t*_*d*_ = 100 ms) and 50 negative voltage pulses (*V*_*D*_ = −1.5 V, *t*_*d*_ = 100 ms) with 100 ms pulse interval. 5,000 consecutive pulses (i.e., 50 cycles) were applied to the PEDOT:PSS/pentacene device. As shown in Figure 3A, the PSC presented a gradual increase/decrease by the train of 50 positive/negative voltage pulses. The non-linearity in update processes of potentiation and depression were calculated to be about 1.51 and 2.20, respectively. The small non-linearity (∼1) can effectively reduce the time redundancy and energy loss in the training process of neural networks (Yu, 2018; Yang F. et al., 2021). The CCV (CCV, i.e., write noise) was designated a significant factor that determines the process of machine learning and the final learning accuracy (Fu et al., 2022). Hence, the probability distribution of the change in channel conductance (Δ*G*) induced by a potentiation or depression pulse was recorded (Figure 3B and Supplementary Figure 9). The statistics were collected from 4,000 switching events. The uniformity of Δ*G* versus *G* determined the write noise of the memristor device, where Δ*G* was the change in conductance due to a “write” operation, and *G*_0_ was the initial conductance state. In our case, the average dynamic variation of Δ*G* was 1.39 μS, and each cycle conductance change to average value was estimated to be as low as 7.91%. An ideal memristor device should have a narrow distribution.

The device non-linearity, noise, and dynamic range of conductance were used for training simulation by CrossSim Simulator (Fuller et al., 2017). We constructed the artificial neural network (ANN) with 784 input neurons, one hidden layer with 1,500 hidden neurons and 10 output neurons. Here, 784 input neurons and 10 output neurons corresponded to the 28 × 28 pixels handwriting numerals recognition Mixed National Institute of Standards and Technology database (MNIST) image dataset and 10 classes of numbers (from 0 to 9). The model was illustrated in Figure 3C. The connection weights of the network were trained by the backpropagation (BP) algorithm numerically (Buscema, 1998). Then, the weight values that fitted by the BP process were readjusted based on the experimental data. As shown in Figure 3D, after 40 iterations of training, the recognition accuracy was up to 92.6%.

### Flexible memristor

**FIGURE 4:**
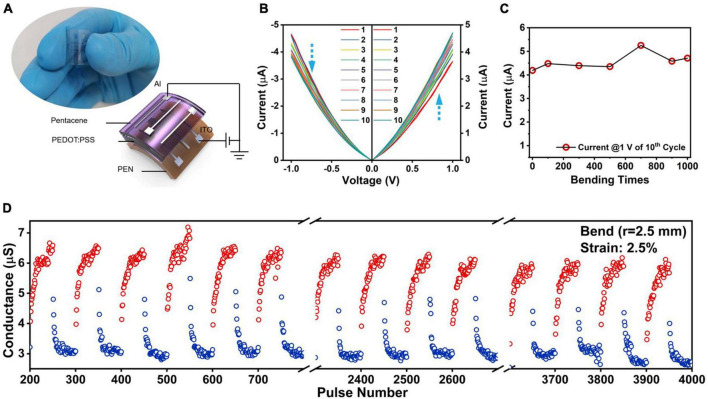
Flexible PEDOT:PSS/pentacene memristor devices. **(A)** Schematic diagram and optical image of the PEN/ITO/PEDOT:PSS/pentacene/Al memristor. **(B)** *I*-*V* curves of the flexible memristor after bending 1,000 times. **(C)** The current value of the 10th sweep cycle of the flexible memristor after bending for different times. **(D)** Potentiation-depression characteristics of the flexible memristor device after 1,000 bending times with 2.5 mm radius. Pulsing scheme: 50 pulse training cycles, with each pulse training cycle comprising 50 identical positive pulses (*V*_P_ = 2.0 V, *t*_d_ = 100 ms, *V*_read_ = 0.5 V) and 50 identical negative pulses (*V*_D_ = –1.5 V, *t*_d_ = 100 ms, *V*_read_ = 0.7 V) with 100 ms pulse interval.

The pretreated PEDOT:PSS solution with GOPS crosslinking agents has been introduced to promote mechanical stability of the films (ElMahmoudy et al., 2017; Ling et al., 2019b). To investigate the mechanical stability of PEDOT:PSS/pentacene RS medium, the flexible organic memristor was fabricated onto ITO-coated poly(ethylene naphthalate) (PEN) substrate and using Al as top electrode, as illustrated in Figure 4A. The *I*–*V* curves obtained after 1,000 tensile bending stress tests with a small bending radius of 2.5 mm was shown in Figure 4B. The flexible memristor device still exhibited reliable analog RS property. The mechanical bending stability as a function of the bending times was shown in Figure 4C. The current values of the 10th sweep cycle were recorded from the same device in the flat state after the selected cycles. The stable current value hinted the good compatibility of PEDOT:PSS/pentacene heterojunction with flexible substrate.

Furthermore, reliable synaptic potentiation and depression behaviors were also demonstrated for flexible PEDOT:PSS/pentacene device (Figure 4C). Even after 1,000 tests of 2.5% stress-strain (Supplementary Figure 12), the variation of training cycles was relatively stable, but with the deterioration of non-linearity (Figure 4D). Using the same simulation model and algorithm, the flexible memristor-based ANN was simulated for handwriting digit recognition training. The relevant data were shown in Supplementary Figures 10, 11. After 40 iterations of training, the recognition accuracy was up to 89.0%, which is only 3.6% lower than that of the rigid memristor-based simulation result. We suggested the degradation was ascribed to the change in the crystal morphology of pentacene and the decrease of effective thickness of PEDOT:PSS film during the bending process, which directly affects its efficiency of carrier transport (Zhou et al., 2012). Therefore, when designing a PEDOT:PSS-based organic heterojunction device, in addition to the matching of energy level, the influence of film morphology on the electrical performance should be further considered, especially for flexible devices.

## Conclusion

In summary, we have fabricated low-power flexible memristor using PEDOT:PSS/pentacene heterojunction. Owing to the higher conductivity PEDOT:PSS film and a well-matched energy level structure of PEDOT:PSS/pentacene, our memristor devices exhibited both low RS operating voltage (1.0 V) and low operating current (< 10 μA), which were lower than those of most reported polymer/organic memristors and were also comparable to that of the state-of-art inorganic memristors (Supplementary Table 1). Various synaptic properties such as potentiation/depression, PPF/PPD, SDDP, SRDP etc., were successfully emulated. A high cycle-to-cycle uniformity was achieved with a low dispersion coefficient (7.91%), enabling the high recognition accuracy approaching 92.6% of MNIST data. Our flexible memristor showed a highly stable memristive effect after sustaining 1,000 tensile bending cycles with a bending radius of 2.5 mm. A high recognition accuracy of 89.0% of MNIST data was simulated using this flexible memristor-based crossbar array. This work paved the way toward a new approach for flexible neuromorphic electronics by using organic heterojunction.

## Materials and methods

### Device fabrication

PEDOT:PSS aqueous suspension (Clevios™ PH1000, Heraeus Electronics Materials) was mixed with ethylene glycol (EG, 5 v/v.%) and dodecyl benzene sulfonic acid (DBSA, 0.1 v/v.%) with (3-glycidyloxypropyl) trimethoxysilane (GOPS, 1 v/v.%). The addition of EG was to increase the film electrical conductivity. The surfactant DBSA was added to facilitate the film processing, also affected film conductivity. The crosslinking agent GOPS was to improve film mechanical stability and adhesion to the substrate. Then the mixed suspension was filtered with a mixed cellulose ester (MCE) membrane (aperture size of 0.8 μm) to remove aggregates for further use. The memristor devices were fabricated on ITO-coated glass slides (1.5 by 1.5 cm). The width of ITO strip electrodes was 100 μm, and thickness of the ITO film was 135 nm with the sheet resistance of 10 Ωcm^–2^. The glass slides were cleaned by acetone, ethanol, and deionized water for 10 min each. Then prepared PEDOT:PSS solution was spin-coated on the ITO/glass substrates with a spin coating rate of 3,000 rpm for 40 s and then annealed in a vacuum oven at 130°C for 60 min. The thin pentacene film was thermal evaporated on PEDOT:PSS under an ultra-slow deposition rate (0.05–0.1 Å/s) for 3 min. The thickness of PEDOT:PSS and pentacene film was 80 and 9 nm, respectively. Finally, 120 nm thick Al top electrode was thermally deposited via shadow mask (strip width of 100 μm).

### Characterizations

The film morphology and thickness were characterized by atomic force microscopy (AFM, Bruker, Dektak XT). *I*-*V* characteristics and synaptic functions were performed with Keithley 2636B source-meter unit at room temperature. External bias was invariably applied to the ITO bottom electrode, while the Al top electrode was grounded.

## Data availability statement

The original contributions presented in this study are included in the article/Supplementary material, further inquiries can be directed to the corresponding authors.

## Author contributions

XL and JM conducted the experimental work and contributed to the writing and editing of the manuscript. JG conducted the ANN simulations. JZ, JF, and ZR analyzed the experimental results. HL and LX conceived and supervised the project. All authors discussed the results.
