# Supplementary material for: Low-power flexible organic memristor based on PEDOT:PSS/pentacene heterojunction for artificial synapse
Source: Front Neurosci. 2022 Sep 8;16:1016026. doi: 10.3389/fnins.2022.1016026 (PMC9492941; doi:10.3389/fnins.2022.1016026)
Supplement: Supplementary file 1 [file Data_Sheet_1.pdf]

Supplementary Material

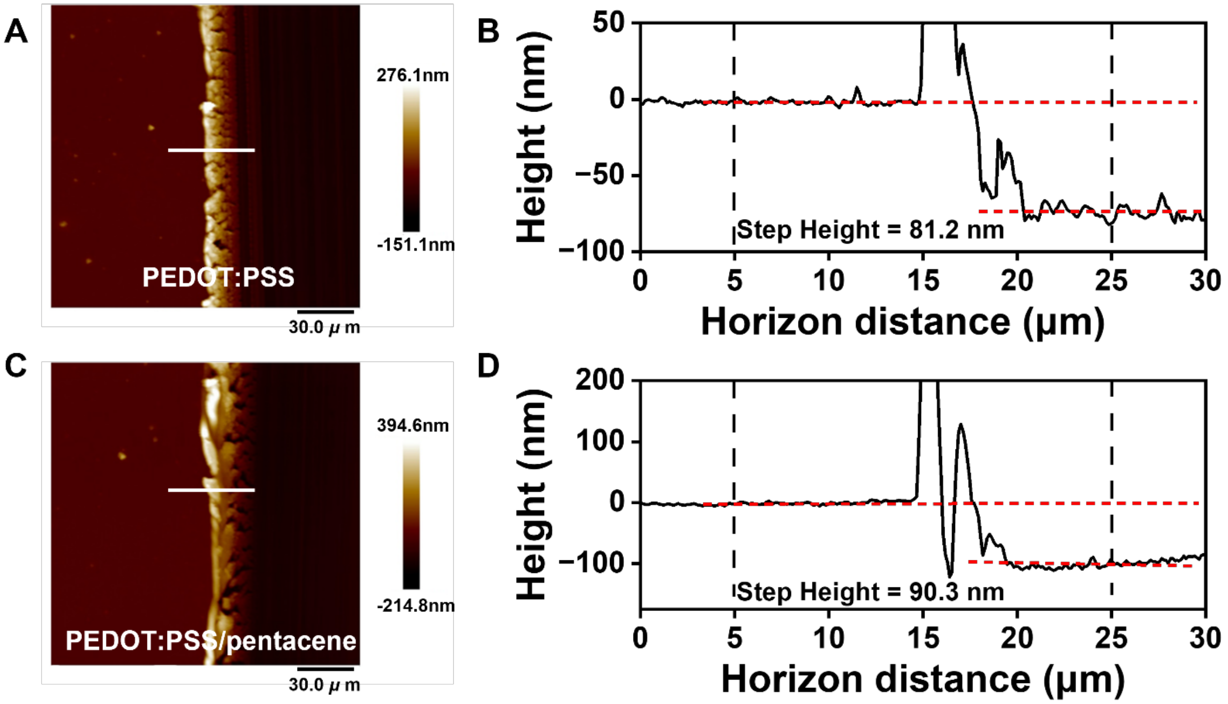

Figure S1. AFM images of PEDOT:PSS film (**A**) and PEDOT:PSS/pentacene film (**C**). (**B**) and (**D**) showed the thickness of the PEDOT:PSS and the whole PEDOT:PSS/pentacene thin film was evaluated by the red dotted line in the figure.

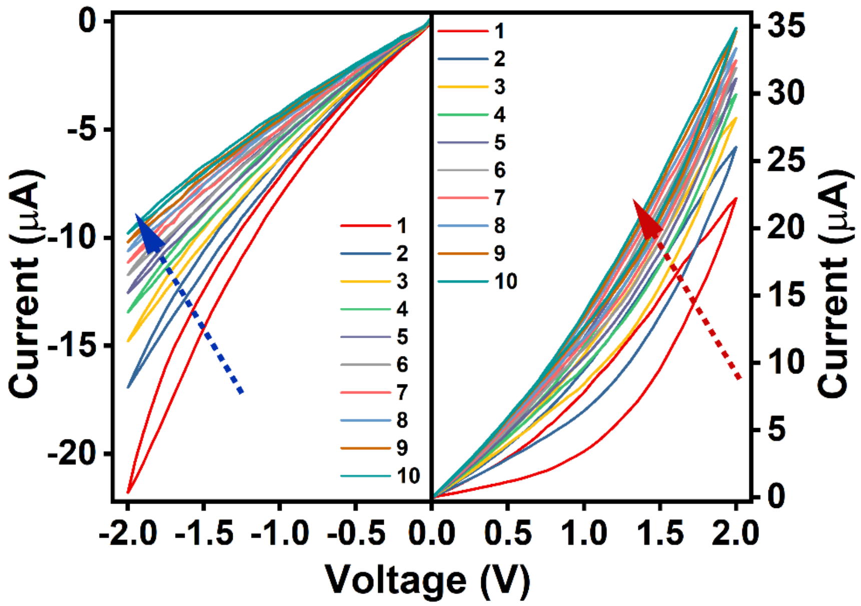

Figure S2. Analog resistive switching properties of the ITO/PEDOT:PSS/pentacene/Al memristor device. 10 times consecutive sweeping cycles (0 V→2.0 V→0 V and 0 V→−2.0 V→0 V, respectively).

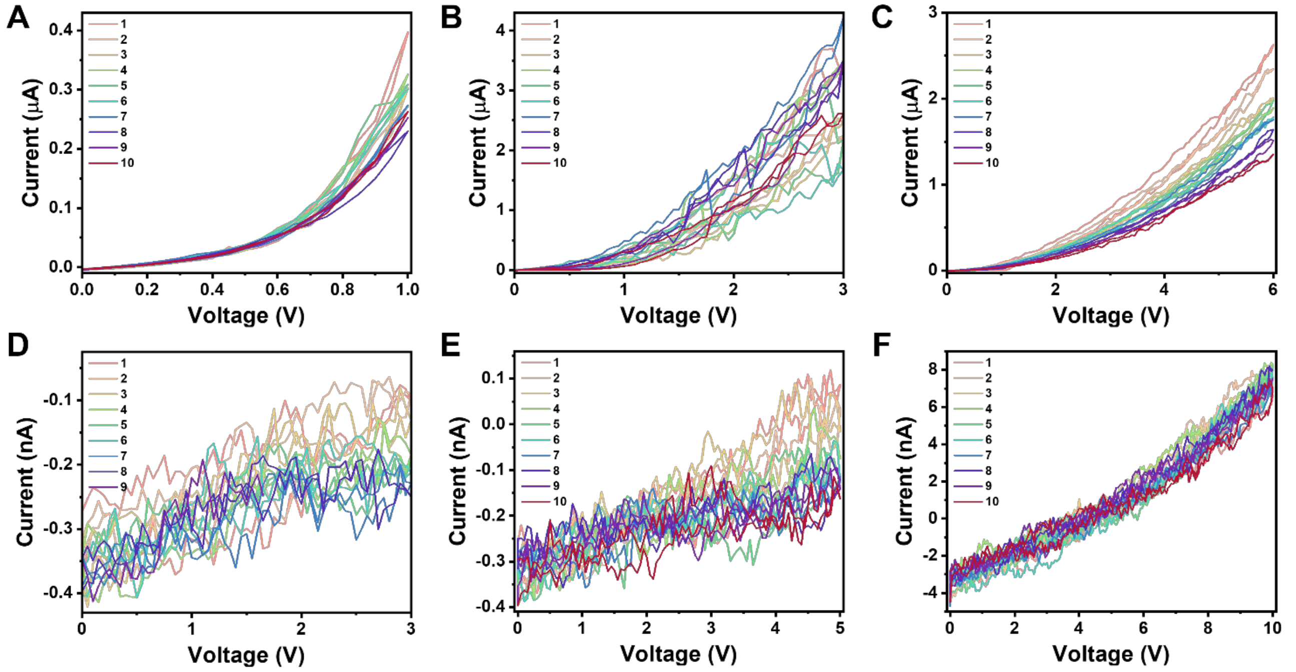

Figure S3. Analog resistive switching properties of the ITO/PEDOT:PSS/Al memristor device with different consecutive sweeping cycles. (**A**) 0 V→1.0 V→0 V, (**B**) 0 V→3.0 V→0 V, (**C**) 0 V→6.0 V→0 V. Analog resistive switching properties of the ITO/pentacene/Al memristor device with different consecutive sweeping cycles. (**D**) 0 V→3.0 V→0 V, (**E**) 0 V→5.0 V→0 V, (**F**) 0 V→10.0 V→0 V.

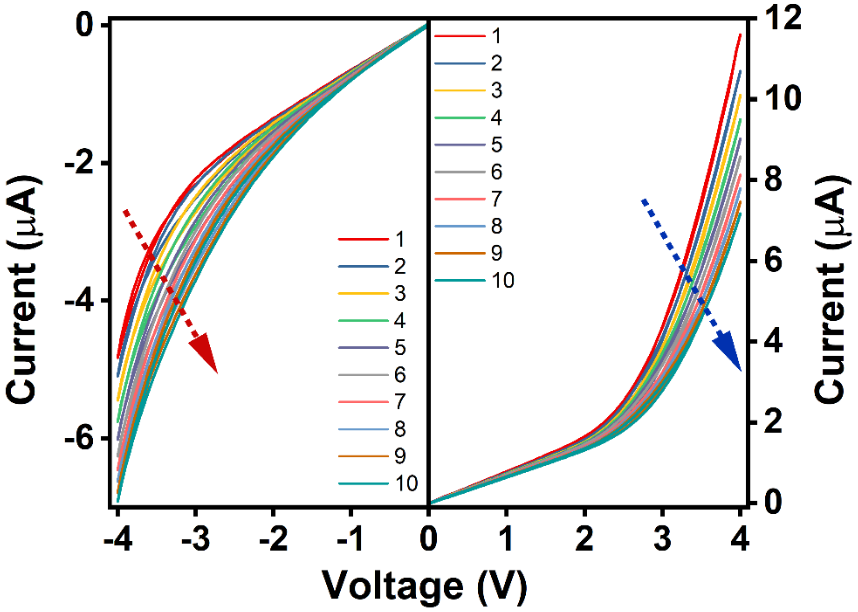

Figure S4. Analog resistive switching properties of the ITO/PEDOT:PSS/pentacene/Al memristor device with the non-addition of additives PEDOT:PSS. 10 times consecutive sweeping cycles (0 V→4.0 V→0 V and 0 V→−4.0 V→0 V, respectively).

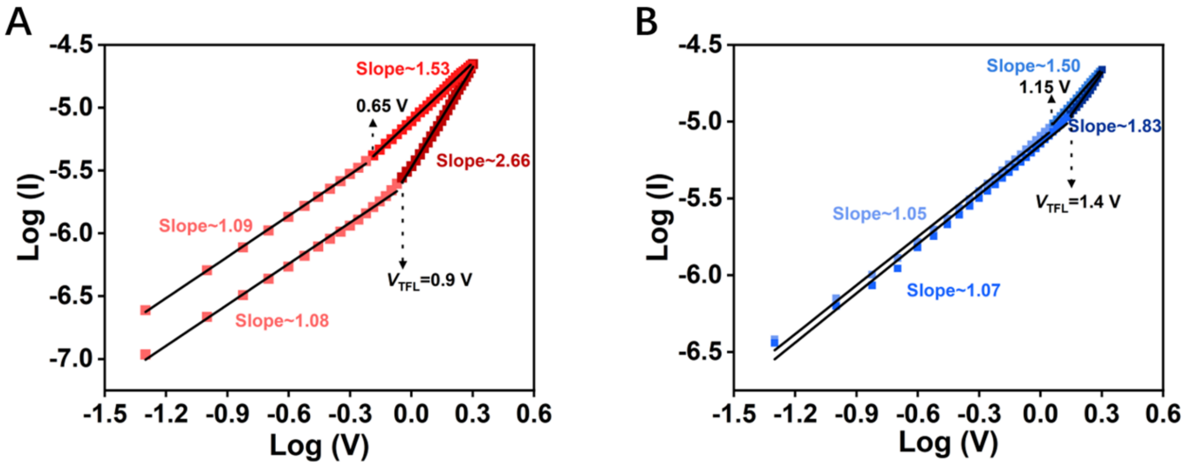

Figure S5. Conduction mechanism analysis. (A) Double logarithmic plot of *I-V* curve (0 V→2.0 V→0 V) in positive voltage region. (B) Double logarithmic plot of *I-V* curve (0 V→−2.0 V→0 V) in negative voltage region.

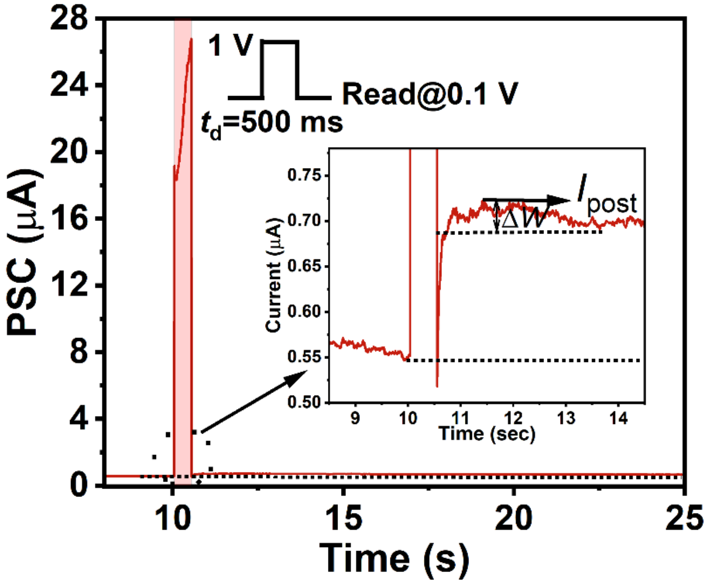

Figure S6. EPSC characteristics triggered by positive voltage pulse (V_P=1.0 V, t_d=500 ms).

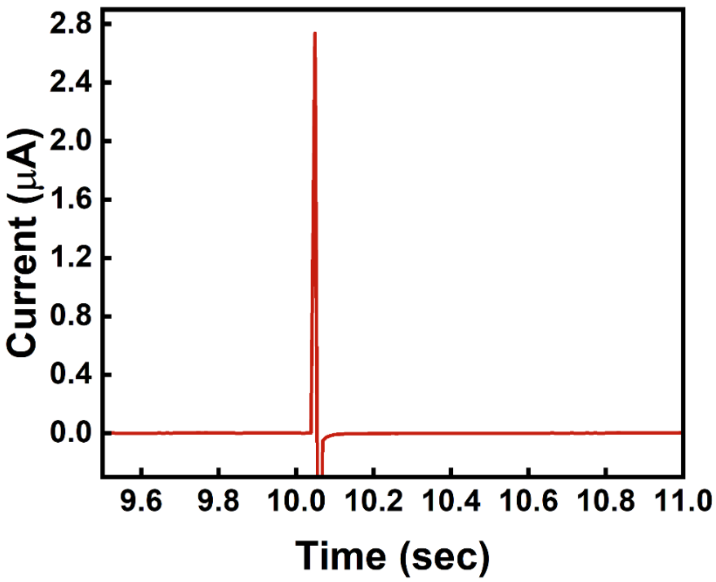

Figure S7. The single synaptic event energy consumption is simply expressed as energy/area: $E = (V \times I \times T) / A$, where E, V, I, T and A are energy consumption, pulse amplitude, peak current, pulse width and cell area, respectively. The energy consumption is estimated to be 2740 fJ μm^{-2} from a peak current of 2.74 μA under a 10 ms pulse at 1.0 V with 10000 μm^2 cell area.

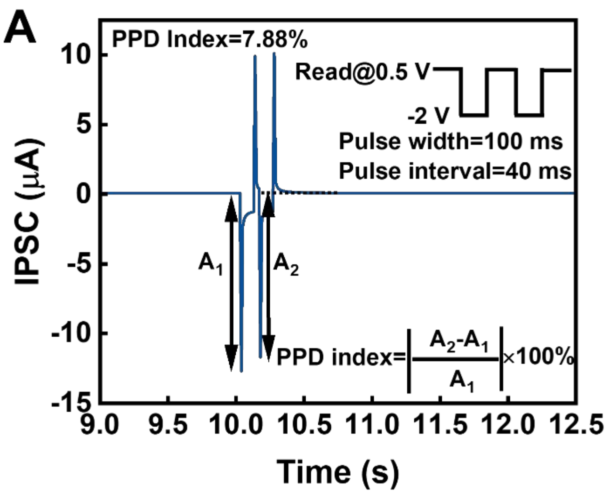

Figure S8. (A) PPD behavior triggered by a pair of negative voltage pulses (V_P=−2.0 V, t_d=100 ms, Δt=40 ms). **(B)** PPF index plotted as a function of pulse interval.

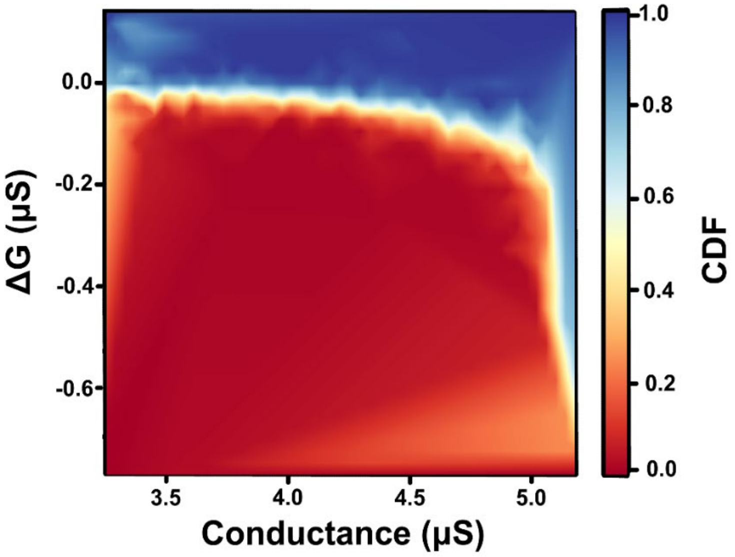

Figure S9. ΔG vs G switching statistics of the rigid PEDOT:PSS/pentacene memristor device during depression. Figure 3b and Figure S9 can determine how much the mean update will be and how much noise to add to that update given the update requested by the algorithm.

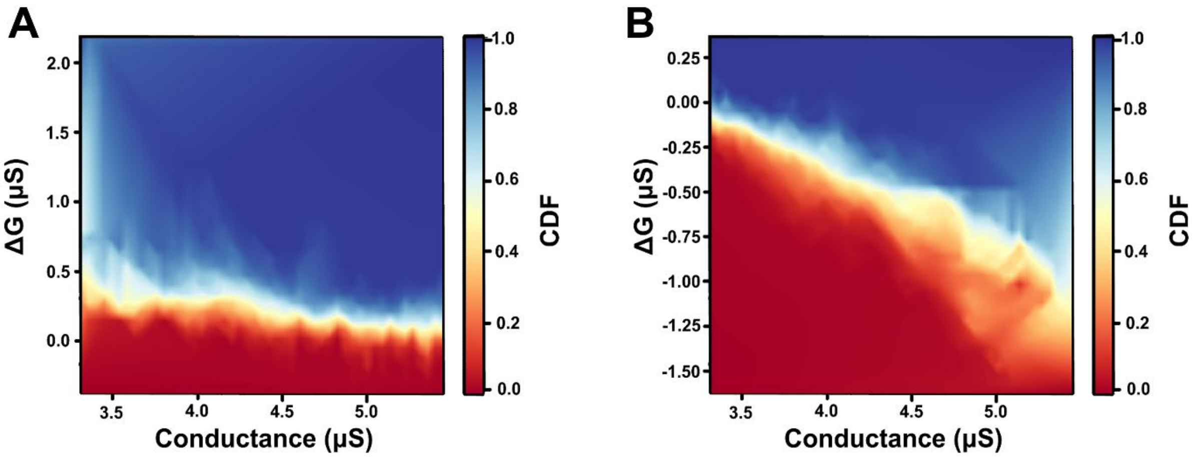

Figure S10. ΔG vs G switching statistics during (**A**) potentiation and (**B**) depression of the flexible PEDOT:PSS/pentacene memristor, respectively.

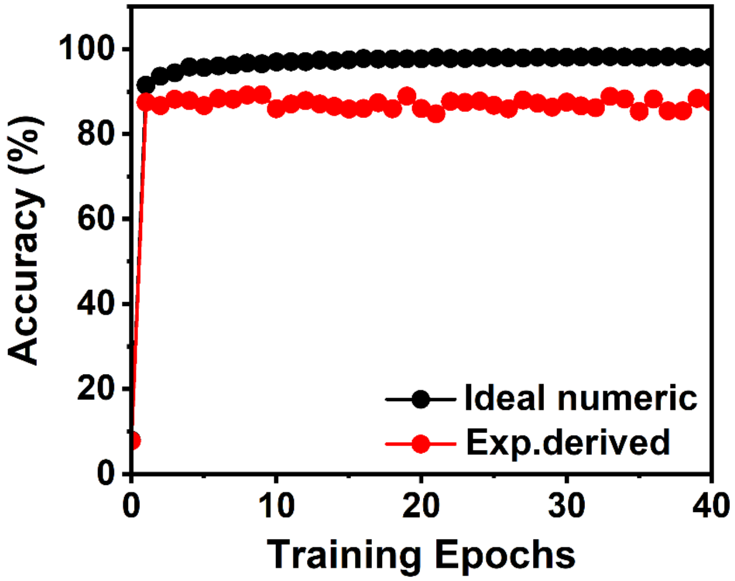

Figure S11. The recognition accuracy evolution with training epochs for handwritten digit image using flexible PEDOT:PSS/pentacene memristor-based crossbar array.

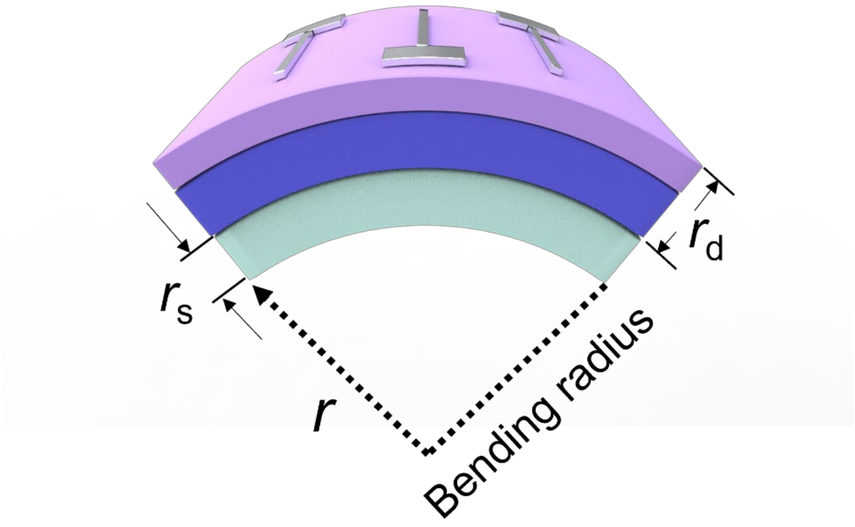

Figure S12. The schematic diagram of the tensile bending orientation with a bending radius (r) of 2.5 mm. The total device thickness is composed of the substrate (t_s) and the device active layer (t_d) thickness. Since the substrate thickness (t_s=0.125 mm) is much larger than the active layer thickness (t_s=90 nm), the substrate thickness is used here to estimate the total device thickness. The stress strain (%) was calculated from the equation below:

$$\varepsilon = \frac{t_s}{2r} \qquad (1)$$

The corresponding stress strain was calculated to be 2.5%.

Table S1. Summary of flexible memristor devices.

Device structure (from top to bottom)	Operating voltage (V)	Single synaptic event energy consumption (cell area)	Bending times	Bending radius (mm)	Neuro-computing based on flexibility	Ref.
Pt/TiO_x/Pt/Parylene-C	1.8/-1.8	2.25×10^5 fJ μm^{-2} (4 μm^2)	-	0.4	-	(Khiat et al., 2016)
Ag/Al_2O_3 NP:PI/ITO/PET	2.5/-4	1.14×10^9 fJ μm^{-2} (220000 μm^2)	400	10	-	(Wu et al., 2017)
Mg/collagen/ITO/PET	3/-3	3.0×10^4 fJ μm^{-2} (10000 μm^2)	-	7	-	(Raeis-Hosseini et al., 2018)
Al/pPPI/ITO	2.4/-1.9	2.49×10^3 fJ μm^{-2} (225 μm^2)	100	20	-	(Ren et al., 2019)
Ag/HZO/ITO/PET	0.99/-1.33	1.60×10^7 fJ μm^{-2} (1000 μm^2)	-	-	-	(Wang et al., 2019)
Al/IAO/In_2O_3/ultratek foil	2/-2	4.42×10^6 fJ μm^{-2} (11304 μm^2)	500	4.2	-	(Zhang et al., 2019)
Al/zein/ITO	0.3/-1.2	3.22×10^5 fJ μm^{-2} (49087 μm^2)	-	3.5	√	(Kim et al., 2021)
Ag/albumen:CuO/ITO/PET	6/-6	3.31×10^5 fJ μm^{-2} (7854 μm^2)	500	16	√	(Guo et al., 2022)
Ag/Mn-ZnO/ITO	2/-2	5.0×10^3 fJ μm^{-2} (100 μm^2)	-	10	-	(Xue et al., 2022)

**ITO/PEDOT:PSS/pentacene/Al**	**1/-1**	**2.74×10^3 fJ μm^{-2} (10000 μm^2)**	**1000**	**2.5**	√	**This work**

References

Guo, T., Ge, J., Sun, B., Pan, K., Pan, Z., Wei, L., et al. (2022). Soft Biomaterials Based Flexible Artificial Synapse for Neuromorphic Computing. *Advanced Electronic Materials* n/a(n/a)**,** 2200449. doi: https://doi.org/10.1002/aelm.202200449.

Khiat, A., Cortese, S., Serb, A., and Prodromakis, T. (2016). Resistive switching of Pt/TiOx/Pt devices fabricated on flexible Parylene-C substrates. *Nanotechnology* 28(2)**,** 025303. doi: 10.1088/1361-6528/28/2/025303.

Kim, Y., Park, C.H., An, J.S., Choi, S.-H., and Kim, T.W. (2021). Biocompatible artificial synapses based on a zein active layer obtained from maize for neuromorphic computing. *Scientific Reports* 11(1)**,** 20633. doi: 10.1038/s41598-021-00076-1.

Raeis-Hosseini, N., Park, Y., and Lee, J.-S. (2018). Flexible Artificial Synaptic Devices Based on Collagen from Fish Protein with Spike-Timing-Dependent Plasticity. *Advanced Functional Materials* 28(31)**,** 1800553. doi: https://doi.org/10.1002/adfm.201800553.

Ren, Y., Chang, C.-L., Ting, L.-Y., Zhou, L., Mao, J.-Y., Zhang, S.-R., et al. (2019). Flexible Pyrene/Phenanthro[9,10-d]imidazole-Based Memristive Devices for Mimicking Synaptic Plasticity. *Advanced Intelligent Systems* 1(1)**,** 1900008. doi: https://doi.org/10.1002/aisy.201900008.

Wang, T.-Y., Meng, J.-L., He, Z.-Y., Chen, L., Zhu, H., Sun, Q.-Q., et al. (2019). Atomic Layer Deposited $Hf_{0.5}Zr_{0.5}O_2$-based Flexible Memristor with Short/Long-Term Synaptic Plasticity. *Nanoscale Research Letters* 14(1)**,** 102. doi: 10.1186/s11671-019-2933-y.

Wu, C., Kim, T.W., Guo, T., Li, F., Lee, D.U., and Yang, J.J. (2017). Mimicking Classical Conditioning Based on a Single Flexible Memristor. *Advanced Materials* 29(10)**,** 1602890. doi: https://doi.org/10.1002/adma.201602890.

Xue, Q., Hang, T., Liang, J., Chen, C.-C., Wu, Y., Ling, H., et al. (2022). Nonvolatile resistive memory and synaptic learning using hybrid flexible memristor based on combustion synthesized Mn-ZnO. *Journal of Materials Science & Technology* 119**,** 123-130. doi: https://doi.org/10.1016/j.jmst.2021.09.007.

Zhang, W., Mao, Y., and Duan, W. (2019). Synaptic and Digital Switching in Diffusion Effect-Assisted Oxides for All-Inorganic Flexible Memristor. *physica status solidi (RRL) – Rapid Research Letters* 13(6)**,** 1900016. doi: https://doi.org/10.1002/pssr.201900016.
